# Integrated Highway Safety Information System (HSIS) datasets by means of a Roadway Safety Data Integrator (RSDI) tool

**DOI:** 10.1016/j.dib.2020.106154

**Published:** 2020-08-07

**Authors:** Seyedehsan Dadvar, Young-Jae Lee, Hyeon-Shic Shin, Hamed Khodaparasti

**Affiliations:** a Transportation Research Engineer, Cyfor Technologies LLC, C/O Geometric Design Laboratory, Turner-Fairbank Highway Research Center, Federal Highway Administration, 6300 Georgetown Pike, McLean, VA 22101, United States; b Department of Transportation and Urban Infrastructure Studies, School of Engineering, Morgan State University, 1700 E. Cold Spring Lane, Baltimore, MD 21251, United States; c City & Regional Planning Program, Department of Graduate Built Environment Studies, School of Architecture and Planning, Morgan State University, 1700 E. Cold Spring Lane, Baltimore, MD 21251, United States; d Software Developer, NorthSoft, Baltimore, MD, United States

**Keywords:** Motor vehicle crash, Accident, Road safety, Highway Safety Manual (HSM), Highway Safety Information System (HSIS), Data integration

## Abstract

The datasets and tool presented in this article are related to the research article entitled “Improving crash predictability of the *Highway Safety Manual* through optimizing local calibration process” (Dadvar et al., 2020) [1], in which these datasets were used to investigate alternative local calibration methods for the *Highway Safety Manual* (HSM) [2] local application. The datasets are integrated Highway Safety Information System (HSIS) [3] state-wide rural two-lane, two-way roads (R2U) data from states of Illinois and Washington.

The HSIS is a database that maintains motor vehicle crash data, roadway inventory, and traffic volume data for several US states. It is an excellent source of data to highway safety research and can be used to investigate many research questions. However, to prepare an analysis-ready roadway safety dataset based on the HSIS or any databases that store the relevant data in multiple different datasets, the researchers should integrate multiple datasets, merge or unmerge and remove certain inconsistent records, and finally clean the integrated dataset. The HSIS staff is usually accommodating and eager to help, but sometimes the nature of data needs is complicated and laborious.

A tool named Roadway Safety Data Integrator (RSDI) was developed for combining, segmenting, and selecting homogeneous (unchanged during the study period for certain variables of interest) HSIS roadway segments and also crash assignment by desired crash attributes (e.g., crash severity or type). The RSDI tool can be helpful for integrating different safety-related datasets such as roadway inventory (including grade, curve, and other subsets), traffic volume, and motor vehicle crash data; also, it can do required segmentation and identify the homogeneous roadway segments over the desired years of study that are the basis for development and calibration of the HSM predictive models.

The shared datasets contain homogeneous roadway segments, geometric details, and crash data for six years from Illinois (2005–10) and Washington (2010–15). The datasets and RSDI tool would be important sources generally for investigating highway safety research questions and in particular, for HSM-related analyses. The RSDI tool can be used for similar purposes and it is not limited to the HSIS data. It can be used for segmentation and finding homogeneous segments of any datasets that follow linear referencing.

The datasets and RSDI tool are hosted in the Mendeley Data repository [4].

**Specifications table**

**Table:** 

**Subject**	Civil and Structural Engineering
**Specific subject area**	Road safety
**Type of data**	Table, image, Excel file, and executable program (.exe)
**How data were acquired**	Raw data was requested from the Highway Safety Information System (HSIS) laboratory of the Federal Highway Administration (FHWA).
**Data format**	Raw and processed
**Parameters for data collection**	The Highway Safety Information System (HSIS) maintains data from several US states; the data availability and quality differ among different states. States of Illinois and Washington were selected due to availability of state-wide curve data which is an important road safety factor.
**Description of data collection**	The raw data was processed using the Roadway Safety Data Integrator (RSDI) tool. While the Illinois dataset included the integrated roadway (including curve data) and crash data, the Washington dataset comprised integrated roadway, curve, grade, lane, and crash data.
**Data source location**	Institution: Highway Safety Information System (HSIS) laboratory, Federal Highway Administration (FHWA) City/Town/Region: McLean, VA Country: USA
**Data accessibility**	Repository name: Mendeley Data (Roadway Safety Data Integrator (RSDI) Tool & Integrated Highway Safety Information System (HSIS) Datasets) Data identification number: DOI: 10.17632/vcj5rzxzw4.1 Direct URL to data: https://data.mendeley.com/datasets/vcj5rzxzw4/1
**Related research article**	S. Dadvar, Y-J. Lee, H-S. Shin, Improving crash predictability of the *Highway Safety Manual* through optimizing local calibration process, Accident Analysis and Prevention 136 (2020). https://doi.org/10.1016/j.aap.2019.105393

**Value of the Data**

• The shared datasets provide state-wide integrated roadway, traffic volumes, and motor vehicle crash data for all rural two-way, two-lane roads in states of Illinois and Washington.
• The datasets are substantial sources in the development of further studies in road safety analysis, crash prediction modeling, and particularly in Highway Safety Manual (HSM) research such as Dadvar et al., 2020 [1].
• Also, the crashes are provided by different severity levels (i.e., fatal (K), injury crashes (A, B, and C), and property-damage-only (O)). Due to importance and impact of fatal and severe injury crashes, the datasets can be used for further severity-based analyses in addition to count-based approaches.
• The shared Roadway Safety Data Integrator (RSDI) tool can be used to reproduce datasets for Illinois, Washington, and other states on the Highway Safety Information System (HSIS). Also, the tool can process any datasets that follow linear referencing.

## 1 Data description

The datasets presented in this data article were used to investigate alternative local calibration methods for the Highway Safety Manual (HSM) local application by Dadvar et al. [1]. The data comprised of integrated Highway Safety Information System (HSIS) datasets (motor vehicle crashes, roadway inventory, and traffic volumes) for all rural two-way, two-lane roads in states of Illinois and Washington (Table 1). The raw data was requested from the Highway Safety Information System (HSIS) laboratory of the Federal Highway Administration (FHWA). States of Illinois and Washington were selected due to availability of state-wide curve data which is an important road safety factor. After receiving the raw data from the HSIS laboratory, the data was processed using the Roadway Safety Data Integrator (RSDI) tool. While the Illinois dataset consists of integrated roadway (including curve data) and crash data, the Washington dataset consists of integrated roadway, curve, grade, lane, and crash data. The description of the variables in integrated datasets are represented in Table 2 and Table 3. The years of data for Illinois and Washington are 2005 through 2010 and 2010 through 2015, respectively. The raw HSIS data (12 Excel files for Illinois including one set of roadway (including curve data) and crash datasets for each year (2005–10) and 30 Excel files for Washington including one set of roadway, curve, grade, lane, and crash datasets for each year (2010–15)), integrated datasets (one Excel file for Illinois and one Excel file for Washington), and RSDI tool and its guide are hosted in the Mendeley Data repository [4].

**Table 1:** Datasets summary.

State	Illinois	Washington
Years	2005 - 10	2010 - 15
Facility Type	Rural two-lane, two-way road (R2U)	Rural two-lane, two-way road (R2U)
Number of Roadway Segments	16,964	40,141
Length	5998.58 miles (9653.78 km)	3505.69 miles (5641.86 km)
Number of Crashes	52,801	17,281
Average Crashes per Mile	8.8	4.9
Average Crashes per KM	5.5	3.1
Integrated HSIS Datasets	Road, Crash	Road, Curve, Grade, Lane, Crash
Number of Variables in Final Dataset	111	123

**Table 2:** Description of variables in Illinois integrated dataset.

Variable	Description	Note
cnty_rte	Road identifier	
Begmp	Begin milepost	
Endmp	End milepost	
ilrdR2U**X**_aadt	AADT	Separate variable for each year (X)
ilrdR2U**X**_county	County	Separate variable for each year (X)
ilrdR2U**X**_curv_rad	Curve radius (ft.)	Separate variable for each year (X)
ilrdR2U**X**_dir_curv	Curve direction	Separate variable for each year (X)
ilrdR2U**X**_lanewid	Lane width (ft.)	Separate variable for each year (X)
ilrdR2U**X**_med_type	Median type	Separate variable for each year (X)
ilrdR2U**X**_outshtp1	Outside shoulder 1 Type	Separate variable for each year (X)
ilrdR2U**X**_outshtp2	Outside shoulder 2 Type	Separate variable for each year (X)
ilrdR2U**X**_outshwd1	Outside shoulder 1 width (ft.)	Separate variable for each year (X)
ilrdR2U**X**_outshwd2	Outside shoulder 2 width (ft.)	Separate variable for each year (X)
ilrdR2U**X**_spd_limt	Speed limit (mi/hr)	Separate variable for each year (X)
ilrdR2U**X**_spln_typ	Special lane type	Separate variable for each year (X)
ilrdR2U**X**_surf_typ	Surface type	Separate variable for each year (X)
ilcrR2U**X**_K	Crash severity K	Separate variable for each year (X)
ilcrR2U**X**_A	Crash severity A	Separate variable for each year (X)
ilcrR2U**X**_B	Crash severity B	Separate variable for each year (X)
ilcrR2U**X**_C	Crash severity C	Separate variable for each year (X)
ilcrR2U**X**_O	Crash severity O	Separate variable for each year (X)

*X* = 2005 through 2010.

**Table 3:** Description of variables in Washington integrated dataset.

Variable	Description	Note
road_inv	Road identifier	
Begmp	Begin milepost	
Endmp	End milepost	
wardR2U**X**_aadt	AADT	Separate variable for each year (X)
wardR2U**X**_county	County	Separate variable for each year (X)
wardR2U**X**_lanewid	Lane width (ft.)	Separate variable for each year (X)
wardR2U**X**_lshl_typ	Left shoulder type	Separate variable for each year (X)
wardR2U**X**_lshldwid	Left shoulder width (ft.)	Separate variable for each year (X)
wardR2U**X**_rshl_typ	Right shoulder type	Separate variable for each year (X)
wardR2U**X**_rshldwid	Right shoulder width (ft.)	Separate variable for each year (X)
wardR2U**X**_surf_typ	Surface type	Separate variable for each year (X)
wardR2U**X**_terrain	Terrain	Separate variable for each year (X)
wacu**X**_curv_max	Curve maximum super-elevation	Separate variable for each year (X)
wacu**X**_curv_rad	Curve radius (ft.)	Separate variable for each year (X)
wacu**X**_dir_curv	Curve direction	Separate variable for each year (X)
wagr**X**_pct_grad	Percent grade	Separate variable for each year (X)
waln**X**_sln_type	Special lane type	Separate variable for each year (X)
wacrR2URd**X**_K	Crash severity K	Separate variable for each year (X)
wacrR2URd**X**_A	Crash severity A	Separate variable for each year (X)
wacrR2URd**X**_B	Crash severity B	Separate variable for each year (X)
wacrR2URd**X**_C	Crash severity C	Separate variable for each year (X)
wacrR2URd**X**_O	Crash severity O	Separate variable for each year (X)
wacrR2URd**X**_U	Crash severity Unknown	Separate variable for each year (X)

*X* = 2010 through 2015.

## 2 Experimental design, materials and methods

**Fig. 1:**
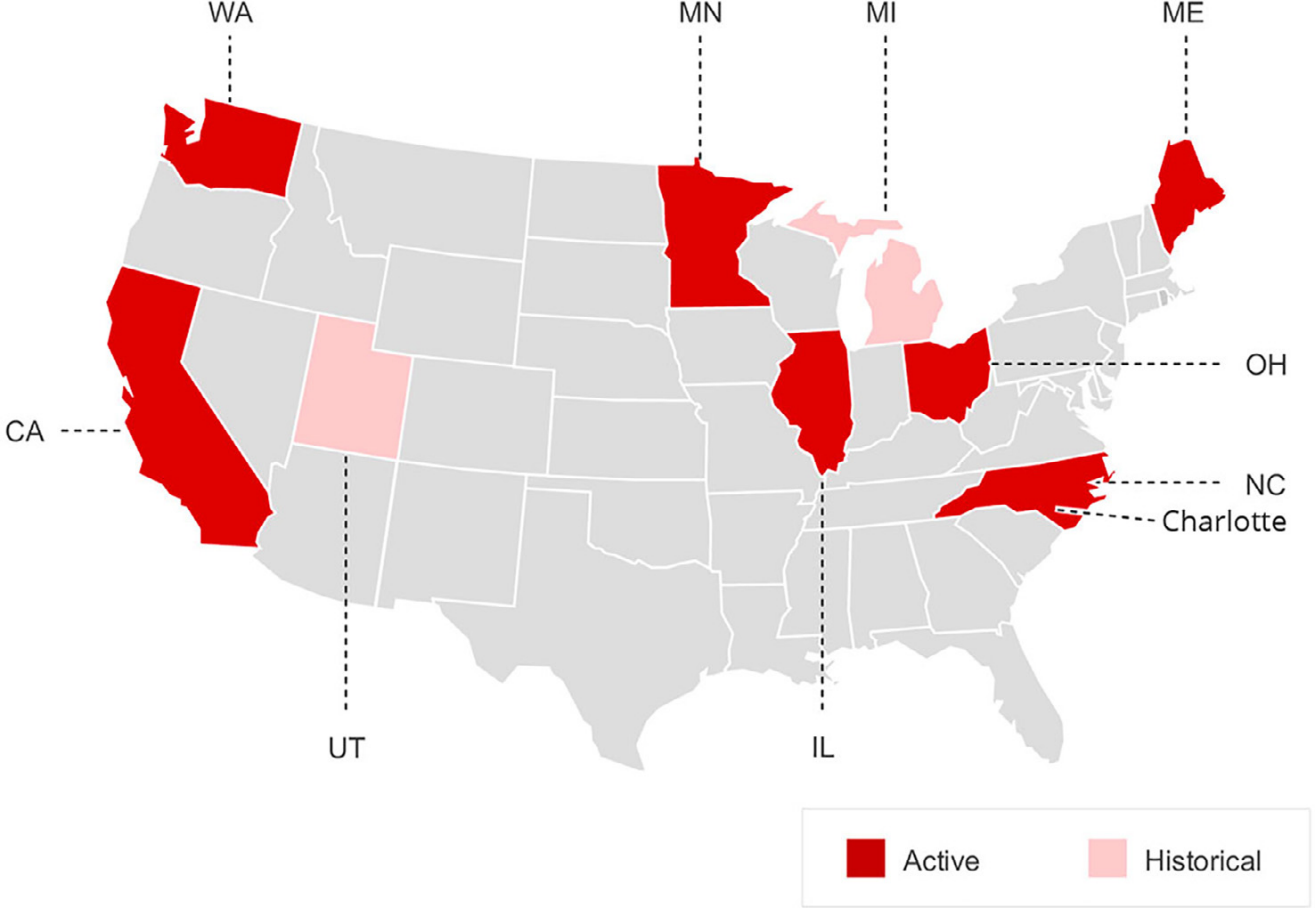
Highway Safety Information System (HSIS): participating states.

Source: [3]

The HSIS is a database that maintains crash data, roadway inventory, and traffic volume data for several different US states (Fig. 1). The HSIS is sponsored by the FHWA and is managed by the University of North Carolina Highway Safety Research Center (HSRC). The HSIS is an appropriate platform to answer many safety-related research questions by historical data from multiple different states. The data for some states are actively being added to the database (such as California, Illinois, and Washington) but historical data is available for Michigan and Utah [3].

### 2.1 Roadway Safety Data Integrator (RSDI) tool

**Fig. 2:**
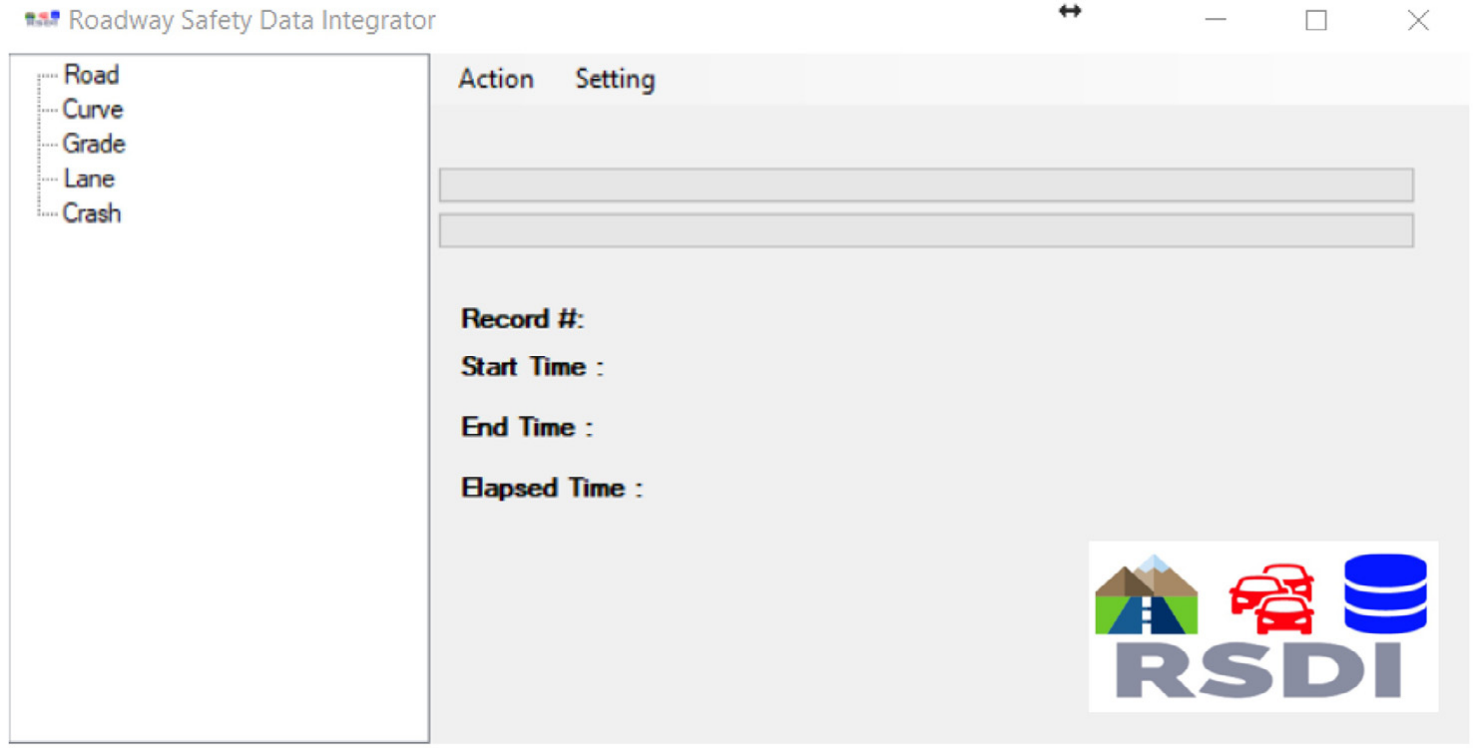
The Road Safety Data Integrator (RSDI) Tool.

To prepare an analysis-ready roadway safety dataset based on the HSIS data or any other databases that store relevant data in multiple different subsets, the researchers should integrate multiple datasets, merge or unmerge and remove inconsistent records, and finally clean the dataset. The HSIS staff is usually accommodating and eager to help researchers, but sometimes the structure of the data needs is complicated and laborious. Also, the application of HSIS data in the HSM analysis requires the records to be homogeneous for multiple years of study; also, crash data (usually by crash severity level) must be assigned to associated sites (roadway segments or intersections) for the desired years of study. The Roadway Safety Data Integrator (RSDI) [5] was developed to avoid the repetitive and laborious data preparation process for different subsets of data or years of study (Fig. 2). The tool was developed in C# language.

The RSDI tool can process a maximum of five different types of datasets for an unlimited number of years such as:

• Roadway geometry and traffic volumes
• Curve data
• Lane data
• Grade data
• Crash data

The required data structure and format to use the RSDI tool are as follows:

• Non-crash data: road inventory id followed by begin MP (milepost) and end MP for roadway geometry, curve, lane, and grade data, then all other variables
• Crash data: road inventory id followed by milepost for each crash, then binary crash data fields
• The same order, number, and names of variables should be used in different years of study.
• The tool works only with CSV format.
• The tool currently works only on roadway segments.

### 2.2 HSIS data preparation

The following steps should be taken on the HSIS raw data before the application of the RSDI tool:

• Sorting the columns alphabetically
• Selecting only the desired roadway facility type(s) roadway segments (e.g., R2U)
• Check, update, and clean the raw data based on:
  ○ Begin MP_i_ < End MP_i_
  ○ Begin MP_i_ < Beg MP_i+1_
  ○ End MP_i_ < End MP_i+1_
  ○ End MP_i_ ≤ Begin MP_i+1_
• Excluding intersection and/or intersection-related crashes based on appropriate crash fields.
• Binary fields for crash data (such as crash severity levels or crash types or combination of them):
  ○ For example, making binary fields for each crash severity level based on KABCO crash severity scale (e.g., if a crash is “fatal” then the binary KABCO scale fields would be: *K* = 1, *A* = 0, *B* = 0, *C* = 0, *O* = 0, *U* = 0)
• AADT estimation (e.g., interpolation) for some roadway segments with missing AADT values

After these steps, the HSIS datasets are ready for identifying homogeneous roadway segments throughout the study period. By the definition and requirement of the HSM [2], the roadway segments should be homogeneous throughout the study period.

### 2.3 Steps of using RSDI tool

The main steps of using the RSDI tool are “add data,” “pre-process,” “process,” “post-process,” and “export” that are explained below.

**Fig. 3:**
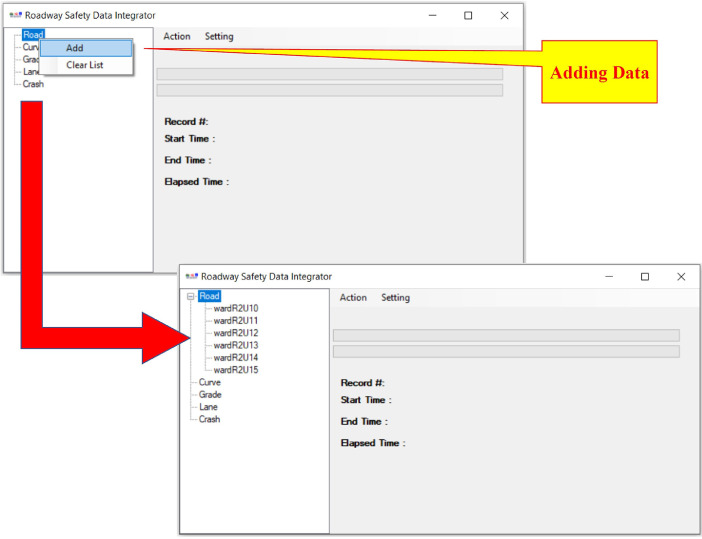
RSDI tool: add raw data.
• Pre-Process (Fig. 4):
  ○ The user selects the fields that should be excluded from finding homogeneous segments (e.g., AADT can differ for homogeneous segments thus it should be excluded from finding homogeneous segments process).
  ○ The tool splits the raw datasets at all required points (either original begin MPs and end MPs or all new points due to the changes in features of interest).

• Add data (Fig. 3):
  ○ The user adds raw datasets (in CSV format) for a maximum of 5 different types of datasets for an unlimited number of years.

**Fig. 4:**
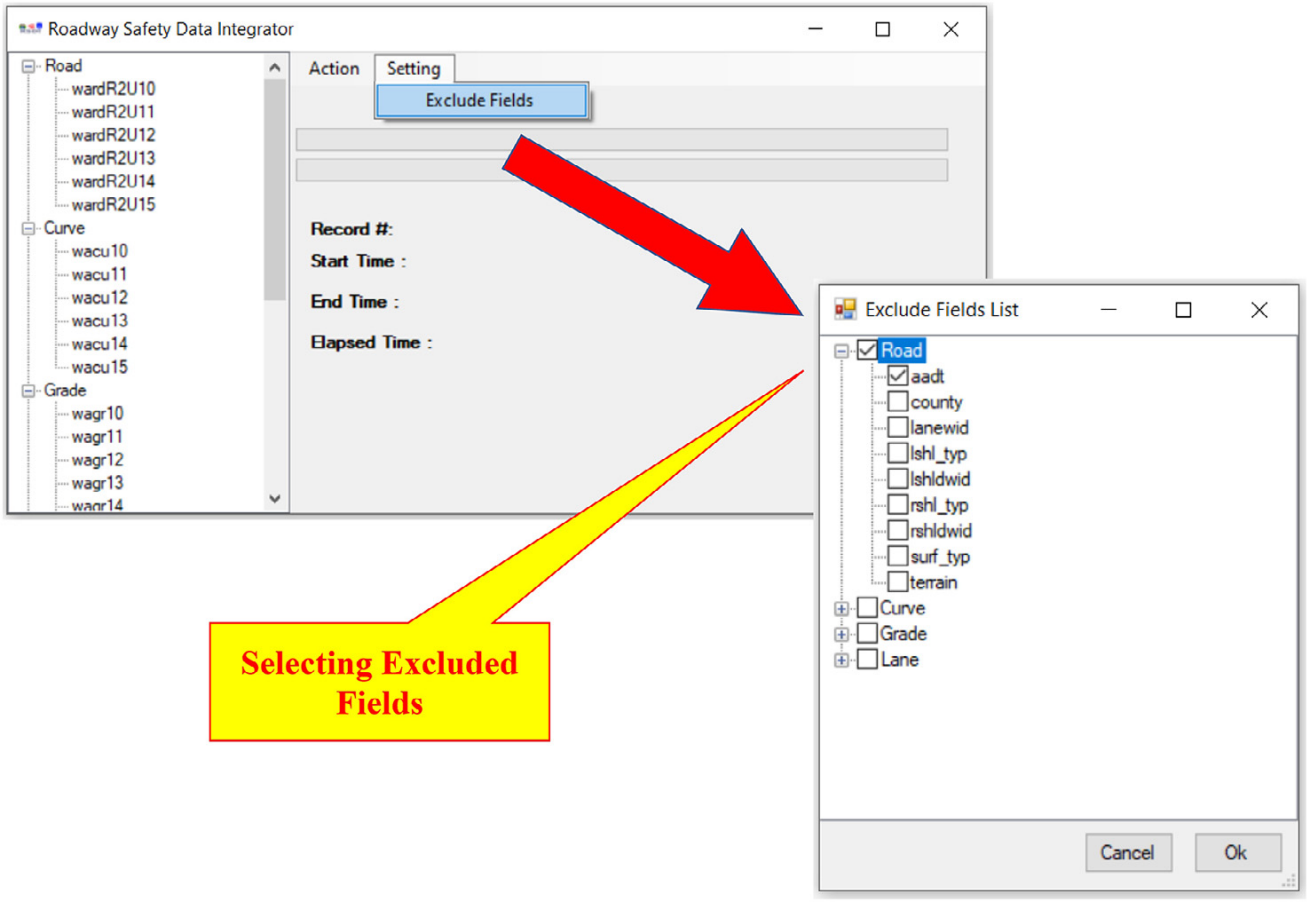
RSDI tool: selecting excluded fields for finding homogeneous roadway segments.
• Process (Fig. 5):
  ○ The tool sorts all datasets based on common field (i.e., road inventory id) and begin MPs and end MPs for all datasets.
  ○ The tool checks the begin MPs and end MPs of all datasets and adds new begin MPs and end MPs to the roadway dataset if need be (Fig. 6).

**Fig. 6:**
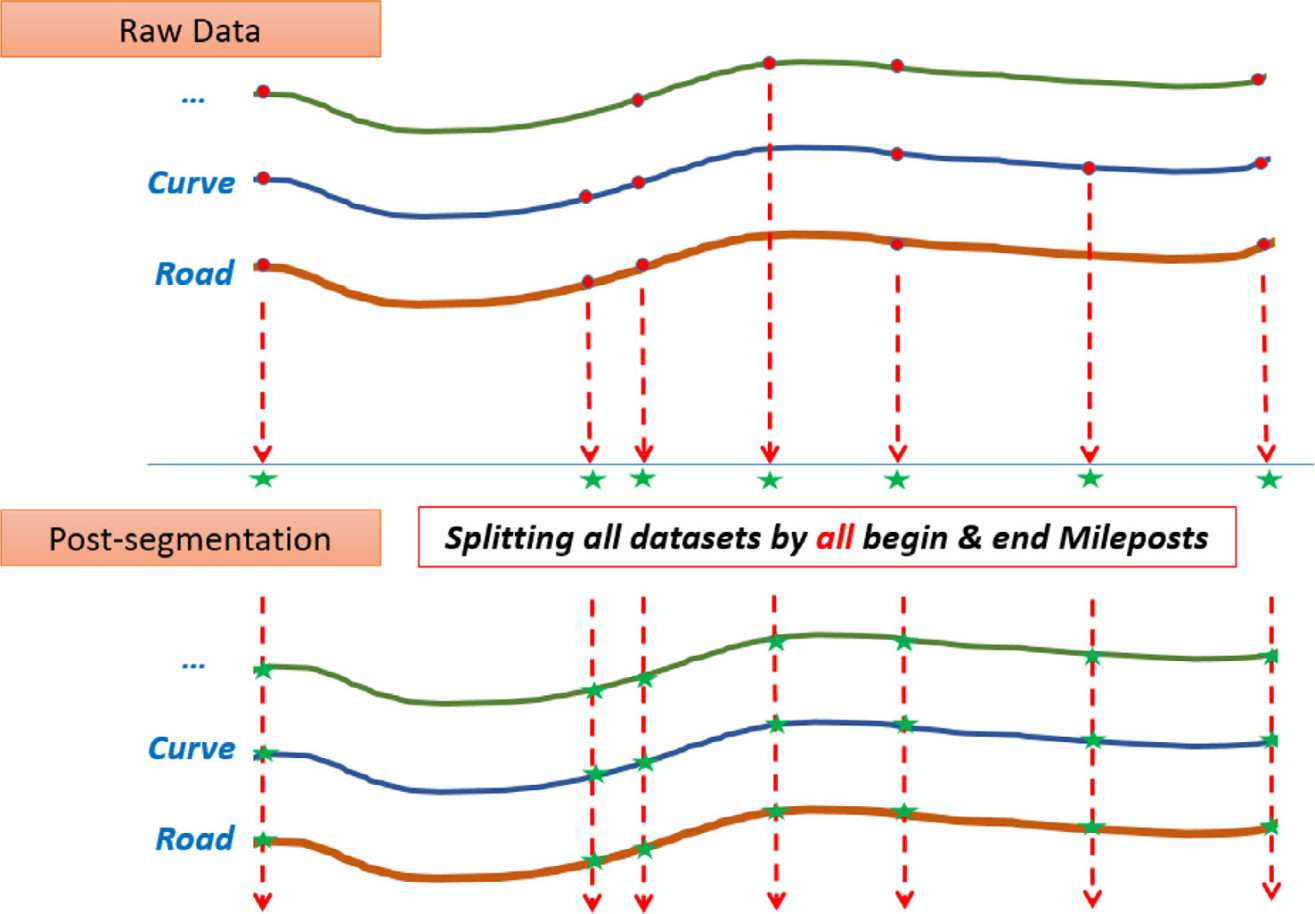
RSDI tool: roadway segmentation.
  ○ The tool combines all datasets together.
  ○ The tool checks if the desired features are consistent (unchanged) throughout the desired years in the combined dataset.
  ○ The tool assigns crash data based on crash MP to the matching roadway segment. If more crashes have occurred on a single roadway segment, the tool adds the number of crashes based on crash filed (e.g., based on crash severity level to the crash severity level fields; KABCO crash severity scale).
  ○ While a crash should be assigned to only one segment, there were some cases that crashes could be assigned to two roadway segments because crashes occurred exactly on the begin MPs and/or end MPs of the roadway segments (e.g., red triangle in Fig. 7). To avoid double crash assignments, all crashes were assigned to the roadway segments that end in those associated mileposts.

**Fig. 7:**
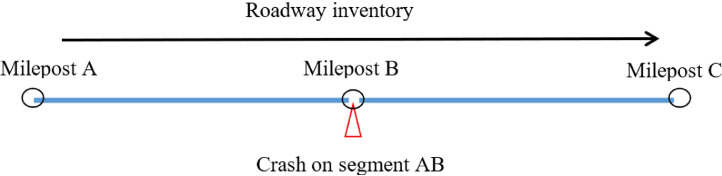
Potential duplication of assigned crashes.
  ○ The tool filters the consistent (unchanged) records during the study period (i.e., homogeneous roadway segments).
  ○ The tool adds notes for the unfiltered records about the first found feature that has changed through the study period.

**Fig. 5:**
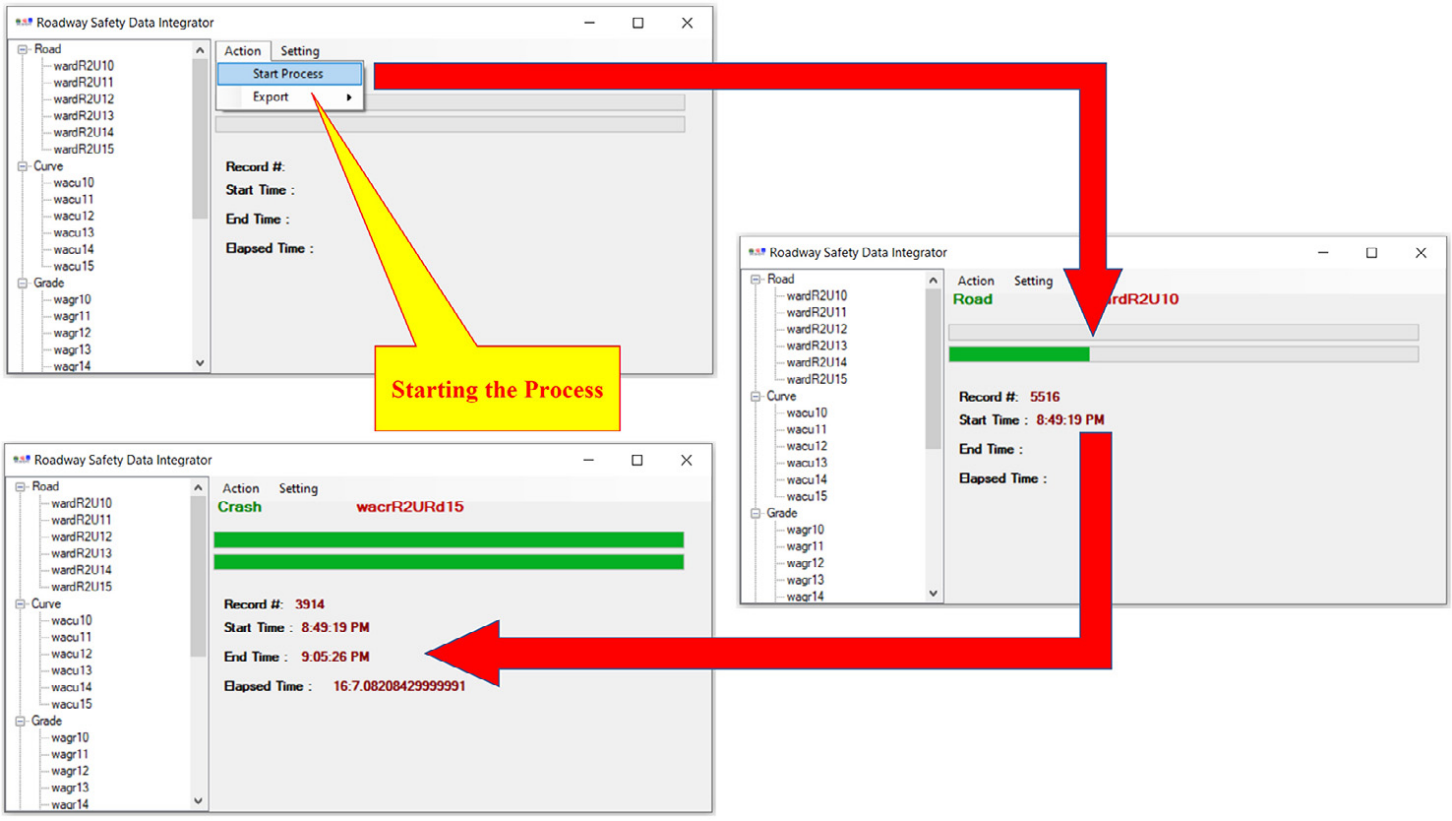
RSDI tool: starting the process.
• Post-Process:
  ○ The tool merges the records with identical features (for example, identical lane width or shoulder type/width) and updates the roadway segment length, begin MP, and end MP, accordingly.
  ○ The tool adds notes for the unmerged records about the first found feature that was not identical to the last record.
• Export (Fig. 8):
  ○ The user has the option to export all and/or filtered (unmerged or merged) final datasets.

**Fig. 8:**
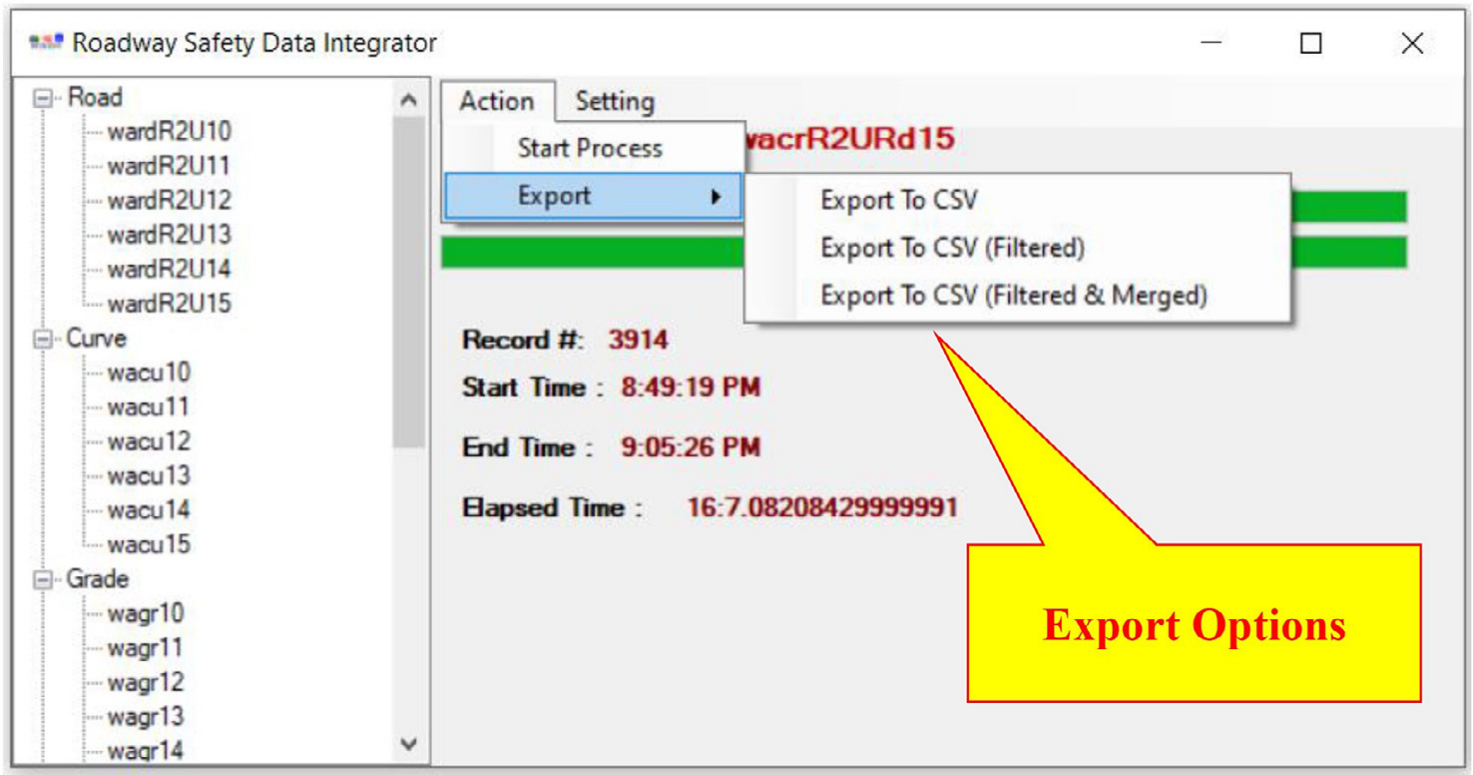
RSDI tool: selecting the export option.

The RSDI tool can be helpful for integrating different safety-related datasets such as roadway inventory (including grade, curve, and other datasets), traffic volume, and crash data; also, it can do required segmentations and identify the homogeneous roadway segments over the desired years of study that are the basis for development and calibration of the HSM predictive models. The RSDI tool can be used for similar purposes and not only limited to the HSIS data. It can be used for segmentation and finding homogeneous segments of any datasets that follow linear referencing. There are avenues for further expansion of the tool; it can currently process five different types of datasets for an unlimited number of years, and making it capable of processing more or unlimited datasets seems a reasonable approach. Moreover, it currently can process only roadway segments, and the inclusion of the intersections requires further research.

## Ethics statement

This work did not involve either human subjects or animal experiments.

## CRediT authorship contribution statement

**Seyedehsan Dadvar:** Conceptualization, Methodology, Data curation, Formal analysis, Investigation, Validation, Writing - original draft, Writing - review & editing. **Young-Jae Lee:** Conceptualization, Methodology, Supervision, Writing - review & editing. **Hyeon-Shic Shin:** Conceptualization, Validation, Supervision. **Hamed Khodaparasti:** Software, Validation.

## Declaration of Competing Interest

The authors declare that they have no known competing financial interests or personal relationships which have, or could be perceived to have, influenced the work reported in this article.

## Appendix Supplementary materials

Image, application 1
